# Effect of supplementation with chokeberry juice on the inflammatory status and markers of iron metabolism in rowers

**DOI:** 10.1186/s12970-014-0048-5

**Published:** 2014-10-01

**Authors:** Anna Skarpańska-Stejnborn, Piotr Basta, Justyna Sadowska, Łucja Pilaczyńska-Szcześniak

**Affiliations:** Department of Morphological and Health Sciences; Faculty of Physical Culture in Gorzów Wlkp, University School of Physical Education in Poznań, 13 Estkowskiego Str., 66 – 400 Gorzów Wlkp, Poland; Department of Water Sports; Branch in Gorzów Wlkp, Faculty of Physical Culture, Gorzów, Poland; School of Humanities, King Stanislaw Leszczynski in Leszno, Leszno, Poland; Department of Hygiene, University School of Physical Education in Poznañ, Poznañ, Poland

**Keywords:** Exercise, Rowers, Supplementation, Chokeberry, Iron, Interleukin

## Abstract

**Background:**

The aim of this study was to analyze the effect of supplementation with chokeberry (*Aronia melanocarpa*) juice on the levels of pro-inflammatory cytokines, hepcidin, and selected markers of iron metabolism in rowers subjected to exhaustive exercise.

**Methods:**

This double-blind study included 19 members of the Polish Rowing Team. The subjects were randomly assigned to the supplemented group (n = 10), receiving 150 mL of chokeberry juice for 8 weeks, or to the placebo group (n = 9). The participants performed a 2000-m test on a rowing ergometer at the beginning and at the end of the preparatory camp. Blood samples were obtained from the antecubital vein prior to each exercise test, one minute after completing the test, and after a 24-hour recovery period. The levels of hepcidin, interleukin 6 (IL-6), tumor necrosis factor alpha (TNF-alpha), ferritin, iron, uric acid, and myoglobin were determined, as well as the total iron-binding capacity, unbound iron-binding capacity, and total antioxidant capacity (TAC).

**Results:**

Post-exercise, there was a significant increase in IL-6 and a significant decrease in the TAC in both groups, prior to and after supplementation with chokeberry juice. At the end of the experiment, the supplemented athletes showed significantly lower post-exercise levels of TNF-alpha and significantly higher TACs and iron levels than the controls.

**Conclusion:**

Supplementation with chokeberry juice results in an increase in the antioxidant activity of plasma and contributes significantly to reducing the TNF-alpha level.

Physical exercise, especially an exhaustive activity, results in a number of unfavorable changes, such as a prooxidant shift in the prooxidant-oxidant balance, hyperthermia, metabolic acidosis, hypoglycemia, and hemoconcentration [1,2]. These processes contribute to a decrease in the osmotic resistance of erythrocytes and their greater susceptibility to hemolysis. This results in an increase in the free iron level, which not only induces free radical-mediated processes, but also enhances inflammation [3,4]. Recent studies showed that inflammatory conditions lead to a decrease in the level of iron, which seems to be analogous to the post-exercise drop off in iron observed in athletes and manifesting as the so-called “sports anemia”. It is likely that this phenomenon is triggered by an acute phase response, which is associated with an increase in the level of cytokines and the resultant enhancement of hepcidin synthesis. The overproduction of hepcidin leads to an accumulation of plasma iron in macrophages and hepatocytes, reduced intestinal absorption of iron by enterocytes, and a decreased plasma level; inevitably, all these processes lead to anemia [5].

The protective role of flavonoids during inflammation may be associated with their ability to sequestrate iron [6] and the regulatory effect they exert on immune components involved in inflammatory processes [7]. An animal model study conducted by Ohgami et al. [8] showed that chokeberry extract exerts strong anti-inflammatory effects during endotoxin-induced uveitis in rats. The authors of this study documented a significant, dose-dependent decrease in the number of inflammatory cells, concentration of protein, and levels of nitric oxide, pyrogenic prostaglandin E_2_ and tumor necrosis factor alpha (TNFα) in the aqueous humor of the chokeberry extract-treated animals. The beneficial effects of chokeberry extract supplementation were also documented in humans [9].

The number of published research documenting anti-inflammatory effects of flavonoids in the context of exercise-induced changes is sparse. Therefore, there is a need to verify if and to what extent, supplementation with polyphenol-rich chokeberry juice may alter the inflammatory indices of elite athletes subjected to intensive exercise load. Moreover, we verified if active compounds of the chokeberry juice may influence the markers of iron metabolism in athletes during the competitive phase of their training cycle. We hypothesized that implementation of the chokeberry juice to athletes’ diet may modulate their post-exercise plasma levels of IL-6 and TNFα, and stimulate favorable changes in iron metabolism parameters.

## Materials and methods

The study included 19 male members of the Polish Rowing Team (16 heavyweight and 3 lightweight rowers). The basic characteristics and sport classes of the athletes are presented in Table 1. The study was conducted between May and July, during an 8-week training camp taking place between the preparatory and competitive phase of a yearly training cycle. The characteristics of a training profile, such as intensity, volume (in minutes), and type (specific, i.e. rowing: endurance, technical, speed, etc., and nonspecific: jogging, strength) were recorded on a daily basis. The intensity of the training was classified in relation to the lactic acid (LA) threshold (4 mmol/L): as an extensive (below the LA threshold) or intensive (above the LA threshold) workload (Table 2).

**Table 1:** **Basic characteristics of the studied groups (mean ± standard deviation)**

**Parameters**	**Supplemented group (n = 10)**	**Control group (n = 9)**
Age (years)	20.5 ± 0.97	20.8 ± 1.09
Body mass (kg)	86.7 ± 1.72	90.2 ± 12.11
Body height (cm)	188.5 ± 1.71	195.8 ± 8.07
Duration of training (years)	5.4 ± 1.1	5.7 ± 1.7

*P* = NS for all between-group comparisons.

**Table 2:** **Training schedules during the weeks preceding blood sample collections before (Trial 1) and after (Trial 2) the supplementation period**

	***Days before trial I***						
	**1**	**2**	**3**	**4**	**5**	**6**	**7**
Total training time, min/day	200	110	210	120	190	110	80
Time rowed, min/day	80	90	110	100	75	90	
Distance rowed, km/day	18	20	24	22	18	20	
Training for force development, min/day	80		80		85		
Extensive endurance rowing training time, min/day	80	60	90	100	30	60	
High intensity endurance rowing training time, min/day		30	20		45	30	
Unspecific training (running, etc.), min/day	40	10	20	20	30	20	80
	***Days before trial II***						
	**1**	**2**	**3**	**4**	**5**	**6**	**7**
Total training time, min/day		90	220	110	170	190	100
Time rowed, min/day		80	130	100	150	110	80
Distance rowed, km/day		16	26	22	30	24	18
Training for force development, min/day			70			60	
Extensive endurance rowing training time, min/day		60	75	100	95	80	60
High intensity endurance rowing training time, min/day			55		65	20	20
Unspecific training (running, etc.), min/day		10	20	10	20	20	20

### Food intake

Throughout the entire study period, the athletes resided and took their meals exclusively at one of the Olympic Games Training Centers. Their regular menu consisted of a mixed diet, providing the recommended dietary allowance (RDA) of carbohydrates, proteins, fats, and micronutrients (vitamins and minerals), as stated in the Polish Nutrition Society guidelines [10]. The athletes’ daily food, caloric, and fruit and vegetable intakes were constant throughout the study period. All the athletes declared that they refrained from drugs, medications, and nutritional supplements for at least two weeks prior to the study and throughout the entire study period.

### Experimental procedure

The athletes who were randomized to the supplemented group (n = 10) received 50 mL of chokeberry juice three times a day for 8 weeks. The anthocyanin content of the chokeberry juice (24 mg/mL) was determined spectrophotometrically. The contents of the other compounds (Table 3) were determined by means of Rapid Resolution Liquid Chromatography (RRLC), using the parameters summarized in Table 4. The controls (n = 9) were given 50 mL of placebo three times a day for 8 weeks. The placebo, which contained a 6.6% solution of betaine [(CH3+)3 N + · CH2COO–] and 1% solution of citric acid, was identical to the chokeberry juice in terms of appearance and taste. Both the chokeberry juice and the placebo were manufactured by Europlant PhytoPharm Klęka S.A. (Poland). To minimize any possible bias caused by subjective factors, both the solutions were placed in identical dark bottles labelled with encoded information on the type of preparation and its recommended dosage. An appropriate measure was affixed to each bottle. The label codes were decoded after completion of the study. All subjects received information about the nature of the investigation and provided their written informed consent to participate in the study. The protocol of the study was approved by the Local Ethics Committee at the Poznań University of Medical Sciences.

**Table 3:** **Content levels of phenolic compounds in extracts from black chokeberry**

**Phenolic compounds**	**mg x L** ^**-1**^
**Chlorogenic acid**	2181.05 ± 48.32
**Rutin**	498.80 ± 8.84
**Caffeic acid**	53.85 ± 0.50
**Ferulic acid**	15.09 ± 0.14
**Quercetin**	117.60 ± 1.45

**Table 4:** **Basic chromatographic parameters**

**Method**	
**Mobile phase A: H**_**2**_ **O: acetic acid (98:2)**	
**Mobile phase B: H**_**2**_ **O: methanol: acetic acid (48:50:2)**	
**Time (min)**	**% B**
0	0
22	80
26	80
28	0
31	0
Flow rate 1.1 mL x min^-1^	

### Training program

Training volumes (expressed in minutes per day) during the weeks preceding the first and the second assessments (referred to as Trial I and Trial II, respectively), which included extensive rowing, intensive rowing, kilometers, and extensive nonspecific training, are shown in Table 2. In the load training phase (before the first assessment), the training volume amounted to 1020 min · wk^−1^, of which approximately 41% was extensive rowing, 21% was nonspecific training such as power training, and the rest was intensive rowing. The total training volume before the second assessment was 880 min · wk^−1^ and comprised approximately 53% extensive rowing, 18% intensive rowing, and 11% land training.

### Rowing performance test

The athletes performed a controlled 2000-m time test on the first day (prior to supplementation) and at the end of the training camp (after supplementation). Each subject had to cover the distance on a rowing ergometer (Concept II, USA) in the shortest time possible. The results of both tests were taken into consideration during the selection to the championship team; therefore, the athletes were well motivated to perform both tests at maximal effort. Before each test, subjects performed a 5-minute individual warm-up.

### Sample treatment

Blood samples were obtained from the antecubital vein, with dipotassium ethylene diamine tetra-acetic acid (K_2_EDTA) used as an anticoagulant. The samples were collected before each 2000-m test (in the morning after an overnight fast), one minute after completing the test, and after a 24-hour recovery period. The samples were centrifuged immediately after obtaining to separate red blood cells from plasma. Serum was frozen immediately and stored at −80°C until use. Additionally, capillary blood samples were obtained from a finger prick before and after each exercise test to assess athletes’ LA levels.

### Measurements

Serum IL-6 was measured using a commercially available enzyme-linked immunosorbent assay (ELISA; Quantikine HS, R&D Systems, Minneapolis, USA) with an assay range of 0.38-10 pg/mL. Serum concentrations of TNF-α (in pg/mL) were quantified using a commercially available enzyme immunoassay (Quantikine, cat. no. DTA00C Human TNF-alpha). Serum hepcidin was measured using a commercially available ELISA kit (Wuhan EIAab Science Co., China) with an assay range of 0.187-12 ng/mL^−1^.

The iron concentration and total iron-binding capacity (TIBC) were determined using the colorimetric method with chromogens (Randox, cat. no. SI257 and TI1010) and the results were expressed in μg/dL. The unsaturated iron-binding capacity (UIBC) was calculated from the formula: UIBC = TIBC – Fe. The myoglobin concentration was determined immunochemically, with an aid of the Myoglobin ELISA kit (Biocom, cat. no. 11170) and the results were expressed in ng/mL. Serum ferritin levels were determined immunochemically, with the aid of a commercially available diagnostic kit (Demeditec, Germany, cat. no. DE7750) and the results were expressed in ng/mL. All the results were adjusted for changes in hematocrit level. The total antioxidant capacity (TAC), considered a marker of the plasma antioxidant capacity, was assessed with a commercially available kit (Cayman, cat no. Antioxidant Assay 709001–96, USA) and the results were expressed as mmol/L. The average intra-assay CV was < 9.0% for all the parameters. The concentration of LA in capillary blood was determined immediately after obtaining the sample, using a commercially available kit (Dr Lange, Germany, cat. no. LKM 140); LA concentrations were expressed in mmol/L.

### Chemicals and reagents

The following reagents were obtained from Sigma: phenolic acids: caffeic, o-coumaric, p-coumaric, ferulic, gallic, and p-hydroxybenzoic; flavonoids: catechin, quercetin, and rutin.

### Chromatography

The RRLC was performed with an Agilent Technologies 1200 series system comprising an autosampler (model G1329B), a pump (model G1312B0), and a diode array detector (model G1315C). The RRLC system was controlled by a ChemStation for LC 3D system. Spectral data from all peaks were recorded from 190–400 nm. Chromatograms were recorded at 280 nm for gallic acid, p-hydroxybenzoic acid, and catechin; at 320 nm for caffeic, p-coumaric, o-coumaric, sinapic, and ferulic acids; and at 360 nm for rutin and quercetin. Compounds were separated on a 50 mm x 4.6 mm, 1.8 μm particle, SB-C18 column (Agilent). This column was thermostatted at 25°C (Table 4).

### Statistical analysis

Statistical analyses were performed using the STATISTICA v. 10.0 software package (StatSoft, Cracow, Poland). All parameters were compared using 2 (supplemented and placebo groups) × 3 (times of measurement) repeated measures analysis of variance (ANOVA). The data distribution was analyzed using the Shapiro-Wilk test. If significant changes were observed in ANOVA tests, Fisher’s *post-hoc* test was applied to locate the source of significant differences. Student’s unpaired *t*-test was used to compare the anthropometric characteristics of the study groups. Except for the rowing time, the results of the 2000-m tests performed prior to and after the supplementation were subjected to intragroup comparisons with Student’s paired *t*-test, and for intergroup comparisons with Student’s unpaired *t*-test. The results achieved during the 2000-m simulated rowing test were subjected to a one-way analysis of variance (ANOVA). Correlations were determined by using the parametric Pearson-R test. Values are reported as the means ± SD. The threshold of statistical significance was set at *P* < 0.05 for all the tests.

## Results

The subjects form the supplemented group and the controls did not differ significantly in terms of mean age, body height, body weight, and years of training (Table 1). The mean power output and total run time during the 2000-m test performed at the beginning of the training camp did not differ between the study groups. Furthermore, no significant differences in the pre- and post-test blood lactate levels were documented when the results for Trial I were compared to those of Trial II (Table 5).

**Table 5:** **Changes in 2,000 m rowing ergometer performance before and after supplementation**

**Parameters**	**Supplemented group (n=10)**		**Control group (n= 9)**	
	**Before**	**After**	**Before**	**After**
Power (watt)	441 ± 30.8	443 ± 34.9	437 ± 35.9	444 ± 38.5
(W x kg^-1^)	5.11 ± 0.23	5.13 ± 0.24	4.88 ± 0.31	4.94 ± 0.29
LA_min_ (mmol x L^-1^)^a^	1.9 ± 0.16	1.8 ± 0.43	1.9 ± 0.12	1.9 ± 0.35
LA_max_ (mmol x L^-1^)^a^	15.2 ± 2.62	15.8 ± 2.09	14.6 ± 2.30	14.8 ± 3.67
Time (s)	370.4 ± 9.58	369.5 ± 10.44	373.8 ± 15.84	371.2 ± 10.33

Values represent the mean ± standard deviation. ^a^LA, lactic acid. There were no significant differences after supplementation relative to before supplementation (*P* < 0.05).

**Figure 1:**
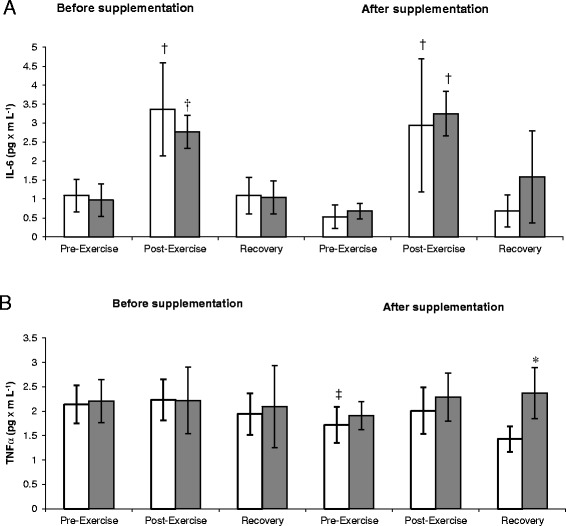
**Changes in interleukin 6 (A) and tumor necrosis factor alpha (B) levels during exercise tests performed before and after the supplementation (mean ±** ***SD*** **).** *Note*: IL-6 = interleukin 6; TNF α = tumor necrosis factor alpha; (gray square) - PLA = placebo group; □ - SUPL = supplemented group; *P < .05 compared to the placebo group; † *P* < .05 compared to the pre-exercise values. ‡ P < .05 compared to the pre-supplementation values.

The pre- and post-test values of IL-6 are presented in Figure 1A. The analysis of variance documented significant exercise-induced changes of this parameter, with no significant effect on the study group. The post-exercise serum level of IL-6 was significantly higher the pre-exercise level, both prior to and after the supplementation period (P < 0.05). In contrast, a significant supplementation effect (main effect *P* < 0.02) and lack of the exercise effect were documented for TNF-alpha levels (Figure 1B). The pre-exercise level of TNF-alpha after supplementation was significantly lower than prior to supplementation. Furthermore, the post-supplementation level of TNF-alpha at recovery was significantly lower than the respective parameter of the placebo group.

**Figure 2:**
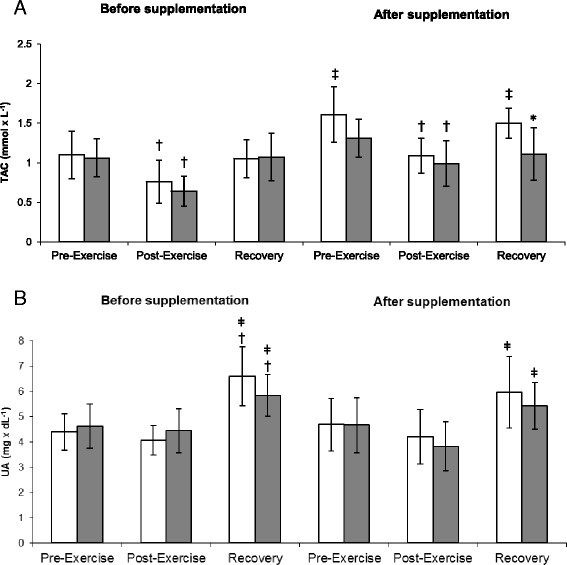
**Changes in the total antioxidant capacity (A) and uric acid (B) levels during exercise tests performed before and after the supplementation (mean ±** ***SD*** **).** *Note*: TAC = total antioxidant capacity; UA = uric acid; (gray square) - PLA = placebo group; **□** - SUPL = supplemented group; *P < .05 compared to the placebo group; † *P* < .05 compared to the pre-exercise values; ǂ *P* < .05 compared to the post-exercise values; ‡ *P* < .05 compared to the pre-supplementation values.

The comparative analysis results of the TAC are presented in Figure 2A. A significant interaction effect of exercise was documented by ANOVA in both groups (*P* < 0.001). Irrespective of the group, the post-exercise TACs were significantly lower than the respective pre-exercise values, both prior to and after supplementation. At the end of the study, the athletes from the supplemented group showed significantly higher TACs at recovery than the individuals from the placebo group. Both prior to and after supplementation, a significant increase in the concentration of uric acid (UA) was observed in both groups when the post-exercise values were compared with the respective levels determined at recovery (Figure 2B).

**Figure 3:**
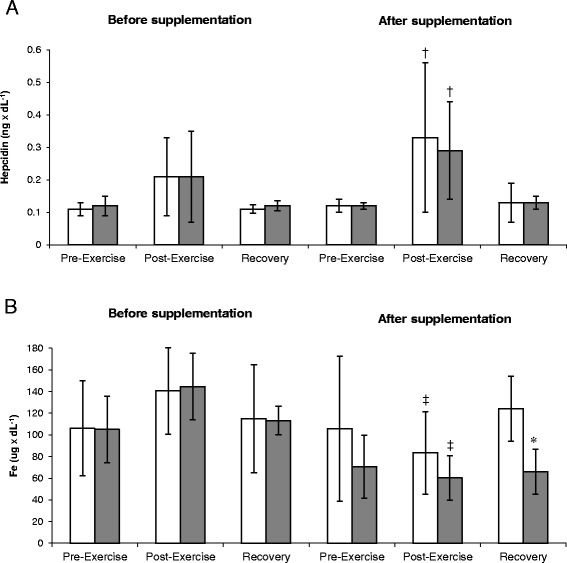
**Changes in hepcidin (A) and serum iron (B) levels during exercise tests performed before and after the supplementation (mean ±** ***SD*** **).** *Note*: Fe = iron; (gray square) - PLA = placebo group; **□** - SUPL = supplemented group; * *P* < .05 compared to the placebo group; † *P* < .05 compared to the pre-exercise values; ‡ *P* < .05 compared to the pre-supplementation values.

Exercise had a significant effect on the hepcidin level by ANOVA (*P* < 0.001). Both groups demonstrated a post-exercise increase in hepcidin level at Trial II (Figure 3A). In addition, the results of ANOVA confirmed that supplementation with chokeberry juice exerted a significant effect on the plasma level of iron (*P <* 0.05). The level of iron determined during the recovery period of Trial II turned out to be significantly higher in the supplemented group than in the controls (Figure 3B). Moreover, physical exercise induced significant changes in the myoglobin levels of the study subjects (main effect *P* < 0.02). A post-exercise increase in myoglobin concentration was observed at Trial I, but not at Trial II (Table 6). No significant interaction effects were documented by ANOVA when comparing the supplementation and exercise groups, with regards to the ferritin, TIBC, and UIBC levels (Table 6).

**Table 6:** **Changes in iron metabolism during exhaustive exercise before and after supplementation**

***Variables***	***Before supplementation***			***After supplementation***			***Exercise***	***P (ANOVA) supplement***	***Exercise × supplement***
	***Pre-exercise x ± SD***	***Post-exercise x ± SD***	***Recovery x ± SD***	***Pre-exercise x ± SD***	***Post-exercise x ± SD***	***Recovery x ± SD***			
Myoglobin (ng x mL^-1^)							0.0129	0.835	0.982
SUPL	203 ± 63.8	346 ± 169.5*	230 ± 68.6	228 ± 168.7	405 ± 283.5	300 ± 110.5			
PLA	216 ± 21.0	318 ± 109.4*	201 ± 39.6	267 ± 192.5	409 ± 340.2	348 ± 233.4			
Ferritin (ng x dL^-1^)							0.062	0.083	0.069
SUPL	74.25 ± 51.18	71.22 ± 29.23	80.00 ± 52.15	80.87 ± 31.02	89.67 ± 33.14	82.44 ± 28.54			
PLA	87.80 ± 27.25	91.00 ± 32.34	87.20 ± 18.58	76.80 ± 24.27	84.00 ± 33.35	77.80 ± 19.25			
TIBC (ug x dL^-1^)							0.087	0.865	0.076
SUPL	320.8 ±32.83	334.2 ± 34.57	325.2 ± 26.82	326.8 ±52.63	339.2 ± 24.37	320.5 ± 31.72			
PLA	316.7 ± 26.36	331.4 ± 25.97	328.3 ± 24.3	330.4 ± 48.3	349.5 ± 48.3	329.8 ± 40.6			
UIBC (ug x dL^-1^)							0.498	0.693	0.735
SUPL	175.0 ± 17.92	189.3 ± 18.87	177.6 ± 14.66	183.5 ± 39.72	194.6 ± 54.87	179.7 ± 38.95			
PLA	179.3 ± 11.62	192.3 ± 28.41	173.4 ± 15.54	190.1 ± 43.34	198.3 ± 49.73	188.43 ± 43.65			

The values represent the mean ±SD. *Abbreviations*: *TIBC* total iron-binding capacity, *UIBC* unsaturated iron-binding capacity, *PLA* placebo group, *SUPL* supplemented group; * *P* < 0.05 relative to pre-exercise - post-exercise.

## Discussion

In this study, we verified the hypothesis that supplementation with chokeberry juice (50 mg three times a day for 6 weeks) may prevent or at least attenuate the consequences of inflammation associated with intensive physical exercise, and exerts beneficial effects on the parameters of iron metabolism. The hereby documented favorable changes after supplementation with the chokeberry juice likely reflected chemical composition of the latter and resultant pleotropic, antioxidative and anti-inflammatory, effects.

The ergometric exercise test performed by our rowers was reflected by a significant post-exercise decrease in the TAC of the plasma, which was observed both prior to and after supplementation (Figure 2A). Previous studies also showed that exhaustive physical exercise can lead to reduction of the plasma TAC [11] and a resultant increase in the concentration of insufficiently neutralized free radicals, which may induce peroxidation of polyunsaturated fatty acids in erythrocyte membranes. Fiorani et al. [12], revealed that human erythrocytes can uptake flavonoids via a passive diffusion mechanism, and, therefore, constitute a specific reservoir. While the vast majority of flavonoids (up to 85%) are accumulated in the cytosol, they are also incorporated into the erythrocyte membrane. According to Arora et al. [13] and Erlejman et al. [14], the flavonoids accumulate at a lipid bilayer-aqueous phase interface, similar to cholesterol and alpha-tocopherol. Due to this intracellular location, flavonoids play vital roles in the stabilization of biological membranes, which become less fluid and thus more resistant to oxidation [15]. It is also worth highlighting the interactions of flavonoids, alpha-tocopherol, and ascorbic acid.

Flavonoids were shown to prevent intracellular oxidation of alpha-tocopherol and convert oxidized alpha-tocopherol back to its radical form (similar to vitamin C). Moreover, flavonoids protect ascorbic acid against oxidative injury and vice versa; thus, the protective effect of flavonoids is enhanced by vitamin C [16,17]. According to Heidi et al. [18], the phenolic compounds present in chokeberry juice are more efficient in regenerating and protecting alpha-tocopherol than ascorbic acid and the phenolic compounds of blackcurrant. These differences were attributed to high concentrations of two anthocyanins, cyanidin-3-arabinoside and cyanidin-3-galactoside, in chokeberry juice and the lack of these compounds in blackcurrants. In turn, Hwang et al. [19], suggested that the strong antioxidant and radical-scavenging activities of black chokeberry extract can be associated with its high levels of antioxidants (total phenolics, total flavonoids, and proanthocyanidin contents), which protect against damage from reactive oxygen radicals. Our study also showed favorable changes in the TAC of athletes who supplemented with chokeberry juice. Compared to the respective pre-supplementation values, a significant increase in the TAC was documented in the supplemented group during the recovery period; furthermore, the post-supplementation TAC determined during the recovery period was significantly higher in the supplemented group than in the controls (Figure 2A).

Braakhuis et al. [20], documented an inverse association between the antioxidant biomarker, the TAC, of rowing athletes and the chronic training dose on a performance test. A similar relationship was also reported by Margonis et al. [21]. The changes in the plasma level of antioxidants, observed after exhaustive physical exercise, are probably associated with a transfer of some of these compounds from tissues to plasma. Previous *in vivo* studies identified uric acid, an end-product of purine metabolism, as a major plasma antioxidant [22]. According to Wayner et al. [23], the uric acid contribution to the TAC of the plasma is about 35-65%. Our athletes showed a significant increase in uric acid concentrations during the recovery periods after the exercise tests performed at Trials I and II (Table 6). However, as mentioned above, the concomitant increase in the TAC was observed solely in the supplemented group.

A relative balance between oxidized, reduced, and radical forms of antioxidants is maintained by flavonoids and constitutes an important element of protection against increased concentrations of reactive oxygen species. However, the role of flavonoids in the chelation of iron ions seems even more important, as this prevents formation of a highly reactive hydroxyl radical, a potent inductor of peroxidation of polyunsaturated fatty acids and polymerization of proteins, which are both present in erythrocyte membranes at high concentrations.

Previous studies [24,25] revealed that structural alterations of erythrocyte membranes, resulting from enhanced generation of free radicals in response to exhaustive or long-term physical exercise, may lead to severe post-exercise hemolysis, which results in an increase in the plasma level of free iron. Under physiological conditions, the iron of heme proteins (hemoglobin, myoglobin, cytochromes) is protected inside a cell. However, it can be released as a result of cellular injury. Therefore, control of free Fe ions represents an important aspect of iron metabolism. Two important biological mechanisms are postulated to be involved in this process. The first is associated with preventing formation of highly toxic reactive oxygen species via the control of free or weakly-bound iron ions, and the second is aimed at protection of iron resources against bacterial degradation [26,27]. A number of bacterial species (e.g. *Mycobacterium tuberculosis, Salmonella* spp., and *Yersinia* spp.) require Fe^3+^/Fe^2+^ ions for their growth. The decrease in pH, associated with exhaustive physical exercise and post-exercise inflammation (secretion of LA by activated granulocytes) promotes release of iron from hemoglobin, ferritin, and transferrin. Therefore, binding Fe^+2^ and other intermediate metals seems to be of vital importance. Anthocyanins, the major component of chokeberry, can chelate iron due to their specific chemical structure (presence of hydroxyl group in the C-ring) [28]. We showed that the same exercise test lead to different effects on the serum concentration of iron. There was an insignificant increase in this parameter at Trial I; while, the concentration slightly decrease at Trial II (Figure 3B). This suggests that the dynamics of serum iron are determined by a phase of training, rather than by the supplementation. We also did not document significant effects of chokeberry juice supplementation on the remaining parameters of iron metabolism, namely the levels of ferritin, TIBC and UIBC (Table 6). Although ferritin is considered an acute phase protein, its level in our rowers did not change significantly after the ergometric test. Similar findings were previously reported by Antosiewicz et al. [29], who found that high-intensity interval exercise (triple Wingate anaerobic test) did not induce statistically significant changes in the levels of ferritin, iron and TIBC of highly trained judo athletes.

The competitive phase of a training cycle (i.e. the period corresponding to the end of our experiment) was characterized by a markedly greater proportion of high-intensity training (Table 4) and a higher severity of post-exercise inflammation. This was also reflected by a post-exercise increase in the TNF-alpha level (Figure 1B). Other authors [30,31] also observed elevated TNF-alpha levels in rowing athletes exposed to intensive training loads.

At the end of our experiment, the levels of iron determined during the recovery period were significantly higher in the supplemented rowers than in the controls (Figure 3B). In addition, we documented a significantly lower pre-exercise level of TNF-alpha in the supplemented group when compared to the pre-supplementation level. Furthermore, the TNF-alpha level at recovery turned out to be significantly lower in the supplemented group than in the controls (Figure 1B). We also observed an inverse correlation between the post-supplementation levels of iron and TNF-alpha (−0.476; p < 0.05). According to a prior report, anthocyanins can attenuate the activity of major inflammatory enzymes, and prevent adhesion of leukocytes and their interaction with vascular endothelial cells via inactivation with TNF-alpha [32]. The administration of blackcurrant extract (equivalent to 48 g of blackcurrants) to individuals performing 30-minutes of exercise on a rowing ergometer with an intensity corresponding to 80% VO_2max_ was reflected by a markedly less pronounced post-exercise increase in TNF-alpha and IL-6 levels [33]. We did not document a significant influence of chokeberry juice on the level of IL-6 (Figure 1A). However, the concentration of this cytokine proved to be significantly modulated by physical exercise, which caused an increase in this parameter, irrespective of the analyzed group and trial. The level of IL-6 was positively correlated with the hepcidin level, both prior to (0.737; p < 0.05) and after the supplementation (0.506; p < 0.05). Hepcidin is considered an acute phase protein, as its synthesis in hepatocytes is induced by IL-6 [34]. This hormone is postulated to be an important mediator of post-exercise iron deficiency, which is observed in response to a number of physiological processes, such as inflammation, hypoxia, and an elevated concentration of Fe resulting from enhanced hemolysis, e.g. due to oxidative injury of erythrocyte membranes [3,35]. We observed a post-exercise increase in the activity of hepcidin solely at Trial II, i.e. after supplementation (Figure 3A). Perhaps, this was the reason behind the post-exercise decreases in the serum level of iron observed after supplementation.

We are well aware of potential limitations of the study. While we measured the parameters of iron metabolism, also determination of the markers of post-exercise hemolysis and its severity, such as bilirubin, haptoglobin, methemalbumin and free hemoglobin, would add considerably to our knowledge of beneficial effects of chokeberry juice in elite athletes.

## Conclusions

We showed that the administration of a natural plant preparation with strong antioxidant potential, chokeberry juice, lead to an increase in the TAC of the plasma, as well as a decrease in the TNF-alpha level during the recovery period. Moreover, we observed a concomitant significant increase in the serum level of iron in the supplemented athletes. These results confirm the beneficial effects of chokeberry juice compounds in reducing the consequences of an intensive training load. Our findings justify the use of chokeberry juice in the supplementation of athletes undergoing maximal exercise.

## Abbreviations

ELISA: Enzyme-linked immunosorbent assay
IL-6: Interleukin 6
LA: Lactic acid
RDA: Recommended dietary allowance
RRLC: Rapid resolution liquid chromatography
TAC: Total antioxidant capacity
TIBC: Total iron-binding capacity
TNF-alpha: Tumor necrosis factor alpha
UIBC: Unsaturated iron-binding capacity

## References

[1] Chatard JC, Mujika II, Guy CC, Lacour JR (1999). Anaemia and iron deficiency in athletes: practical recommendations for treatment. Sports Med.

[2] Reeder B, Wilson M (2001). The effects of pH on the mechanism of hydrogen peroxide and lipid hydroperoxide consumption by myoglobin: a role for the protonated ferryl species. Free Radic Biol Med.

[3] Peeling P, Dawson B, Goodman C, Landers G, Trinder D (2008). Athletic induced iron deficiency: new insights into the role of inflammation, cytokines and hormones. Eur J Appl Physiol.

[4] Peeling P, Dawson B, Goodman C, Landers G, Wiegerinck E, Swinkels D, Trinder D (2009). Training surface and intensity: inflammation, hemolysis, and hepcidin expression. Med Sci Sports Exerc.

[5] Kong W, Gao G, Chang Y (2014). Hepcidin and sports anemia. Cell Biosci.

[6] Seeram NP, Nair MG (2002). Inhibition of lipid peroxidation and structure-activity-related studies of the dietary constituents anthocyanins, anthocyanidins, and catechins. J Agric Food Chem.

[7] Qin B, Anderson R (2012). An extract of chokeberry attenuates weight gain and modulates insulin, adipogenic and inflammatory signalling pathways in epididymal adipose tissue of rats fed a fructose-rich diet. Br J Nutr.

[8] Ohgami K, Ilieva I, Shiratori K, Koyama Y, Jin X, Yoshida K, Kase S, Kitaichi N, Suzuki Y, Tanaka T, Ohno S (2005). Anti-inflammatory effects of aronia extract on rat endotoxin-induced uveitis. Invest Ophthalmol Vis Sci.

[9] Naruszewicz M, Laniewska I, Millo B, Dłuzniewski M (2007). Combination therapy of statin with flavonoids rich extract from chokeberry fruits enhanced reduction in cardiovascular risk markers in patients after myocardial infraction (MI). Atherosclerosis.

[10] Ziemlański S: **Norm of the man’s nutrition.** Warszawa: PZWL; 2001.

[11] Groussard CC, Rannou BF, Machefer GG, Chevanne MM, Vincent SS, Sergent OO, Cillard J, Gratas DA (2003). Changes in blood lipid peroxidation markers and antioxidants after a single sprint anaerobic exercise. Eur J Appl Physiol.

[12] Fiorani M, Accorsi A, Cantoni O (2003). Human red blood cells as a natural flavonoid reservoir. Free Radic Res.

[13] Arora AA, Strasburg GM, Nair MG, Byrem TM (2000). Modulation of liposomal membrane fluidity by flavonoids and isoflavonoids. Arch Biochem Biophys.

[14] Erlejman A, Verstraeten S, Fraga C, Oteiza P (2004). The interaction of flavonoids with membranes: potential determinant of flavonoid antioxidant effects. Free Radic Res.

[15] Bonarska-Kujawa D, Pruchnik H, Oszmiański J, Sarapuk J, Kleszczyńska H (2011). Changes caused by fruit extracts in the lipid phase of biological and model membranes. Food Biophys.

[16] Clemetson CB: **Vitamin C/author, C. Alan B. Clemetson.** Boca Raton, Fla: CRC Press; 1989.

[17] Block G, Henson D, Levine M (1991). Vitamin C: a new look. Ann Intern Med.

[18] Heidi G, Eleonora B, Leif S, Mogens A (2008). Antioxidant synergism between fruit juice and α -tocopherol. A comparison between high phenolic black chokeberry (Aronia melanocarpa) and high ascorbic blackcurrant (Ribes nigrum). Eur Food Res Technol.

[19] Hwang S, Yoon W, Lee O, Cha S, Kim J (2014). Radical-scavenging-linked antioxidant activities of extracts from black chokeberry and blueberry cultivated in Korea. Food Chem.

[20] Braakhuis A, Hopkins W, Lowe T (2014). Effects of dietary antioxidants on training and performance in female runners. Eur J Sport Sci.

[21] Margonis K, Fatouros I, Jamurtas A, Nikolaidis M, Douroudos I, Chatzinikolaou A, Mitrakou A, Mastorakos G, Papassotiriou I, Taxildaris K, Kouretas D (2007). Oxidative stress biomarkers responses to physical overtraining: implications for diagnosis. Free Radic Biol Med.

[22] Kaur H, Halliwell B (1990). Action of biologically-relevant oxidizing species upon uric acid. Identification of uric acid oxidation products. Chem Biol Interact.

[23] Wayner D, Burton G, Ingold K, Barclay L, Locke S (1987). The relative contributions of vitamin E, urate, ascorbate and proteins to the total peroxyl radical-trapping antioxidant activity of human blood plasma. Biochim Biophys Acta.

[24] Aaseth J, Birketvedt G (2012). Hemolysis and rhabdomyolysis after marathon and long distance running. Immunol End Metabol Agents – Med Chem.

[25] Kobayashi Y, Nakatsuji A, Aoi W, Wada S, Kuwahata M, Kido Y (2010). Intense exercise increases protein oxidation in spleen and liver of mice. Nutr Metab Insights.

[26] Cisowska A, Wojnicz D, Hendrich A (2011). Anthocyanins as antimicrobial agents of natural plant origin. Nat Prod Commun.

[27] Ward R, Crichton R, Taylor D, Della Corte L, Srai S, Dexter D (2011). Iron and the immune system. J Neural Transm.

[28] Hider R, Liu Z, Khodr H (2001). Metal chelation of polyphenols. Methods Enzymol.

[29] Antosiewicz J, Kaczor J, Kasprowicz K, Laskowski R, Kujach S, Luszczyk M, Radziminski L, Ziemann E (2013). Repeated "all out" interval exercise causes an increase in serum hepcidin concentration in both trained and untrained men. Cell Immunol.

[30] Rämson R, Jürimäe J, Jürimäe T, Mäestu J (2008). The influence of increased training volume on cytokines and ghrelin concentration in college level male rowers. Eur J Appl Physiol.

[31] Main L, Dawson B, Grove J, Landers G, Goodman C (2009). Impact of training on changes in perceived stress and cytokine production. Res Sports Med.

[32] Speciale A, Virgili F, Cimino F, Saija A, Canali R, Chirafisi J (2010). Cyanidin-3-O-glucoside Protection against TNF-Îł-Induced Endothelial Dysfunction: Involvement of Nuclear Factor-ÎðB Signaling. J Agric Food Chem.

[33] Lyall KA, Hurst SM, Cooney JJ, Jensen DD, Lo KK, Hurst RD, Stevenson LM (2009). Short-term blackcurrant extract consumption modulates exercise-induced oxidative stress and lipopolysaccharide-stimulated inflammatory responses. Am J Physiol Regul Integr Comp Physiol.

[34] Nemeth E, Valore E, Territo M, Schiller G, Lichtenstein A, Ganz T (2003). Hepcidin, a putative mediator of anemia of inflammation, is a type II acute-phase protein. Blood.

[35] Nemeth E, Rivera S, Gabayan V, Keller C, Taudorf S, Pedersen BK, Ganz T (2004). IL-6 mediates hypoferremia of inflammation by inducing the synthesis of the iron regulatory hormone hepcidin. J Clin Invest.

